# Protective Effects of *Artemisia sphaerocephala* Krasch Polysaccharide Against Polyethylene-Microplastic-Induced Intestinal and Testicular Injury in Male Mice

**DOI:** 10.3390/nu18193224

**Published:** 2026-09-29

**Authors:** Peng Chen, Wenting Li, Yujia Li, Chunhua Zhang, Hua Bao, Haizhou Sun

**Affiliations:** 1 College of Animal Science, Inner Mongolia Agricultural University, Hohhot 010018, China; chensky9995@163.com; 2 Institute of Animal Nutrition and Feed, Inner Mongolia Academy of Agricultural & Animal Husbandry Sciences, Hohhot 010031, China; wuclwt@126.com (W.L.); 15164786946@163.com (Y.L.); zhangch-602@163.com (C.Z.); baohua009@126.com (H.B.); 3 Key Laboratory of Grass-Feeding Livestock Healthy Breeding and Livestock Product Quality Control (Co-Construction by Ministry and Province), Ministry of Agriculture and Rural Affairs, Hohhot 010031, China; 4 Inner Mongolia Key Laboratory of Herbivore Nutrition Science, Hohhot 010031, China; 5 Inner Mongolia Autonomous Region Industrial Technology Engineering Center for Product Quality Regulation of Livestock Products, Hohhot 010031, China

**Keywords:** *Artemisia sphaerocephala* Krasch polysaccharide, polyethylene microplastics, gut microbiota, testicular injury, inflammatory response, antioxidant capacity

## Abstract

Background: Microplastics can cause intestinal flora imbalance and testicular damage. *Artemisia sphaerocephala* Krasch polysaccharide (ASKP) is a plant-derived polysaccharide with antioxidant activity and immunomodulatory properties, but the protective effects of ASKP against polyethylene-microplastic-induced intestinal and testicular injury remain unclear. This study investigated the effects of ASKP on intestinal and testicular damage in male mice induced by polyethylene microplastics (PE-MPs). Methods: Sixty male ICR mice were randomly assigned to five groups: untreated mice (control group, CON); PE-MP-only-treated mice (MP group); and PE-MP-exposed mice orally administered with a crude ASKP preparation at 150, 300, and 450 mg/kg BW (LAM, MAM, and HAM groups, respectively) for 60 days. Results: PE-MP exposure reduced the average daily gain, jejunal villus height, and serum testosterone and follicle-stimulating hormone levels while increasing inflammatory responses and oxidative stress levels. However, ASKP supplementation, particularly at 300 mg/kg, partially restored growth performance, intestinal villus morphology, antioxidant capacity, anti-inflammatory cytokine levels, sex hormone concentrations and seminiferous tubule diameter. Gut microflora analysis showed that crude ASKP preparation increased the *Anaerotruncus*, *Mucispirillum* and *Candidatus Arthromitus* abundances while reducing *Desulfovibrio* abundance. Spearman correlation analysis revealed significant associations between these microbial taxa and inflammatory/oxidative stress markers. Conclusions: Crude ASKP preparation mitigates PE-MP-induced toxicity in male mice by reducing oxidative stress and inflammation levels and improving gut microbiota composition and reproductive hormone levels.

## 1. Introduction

With the widespread use of plastic products, the problem of plastic waste has become increasingly serious. The current annual production of plastic waste worldwide is 35 million tons. It is projected that by 2040, the production of plastic waste will soar to around 250 million tons [1]. Under chemical, physical, and biological weathering forces, plastic waste gradually degrades into particles, fibers, or fragments with a diameter of ≤5 mm, termed microplastics (MPs) [2]. MPs have become emerging environmental contaminants owing to their widespread distribution in terrestrial and aquatic ecosystems [3]. Polyethylene (PE), one of the most extensively produced and utilized polymers worldwide, is frequently detected in food, drinking water, and air in the form of polyethylene microplastics (PE-MPs), resulting in unavoidable exposure in both humans and animals [4]. Growing evidence has suggested that ingested MPs can accumulate in multiple organs and induce oxidative stress, inflammation, metabolic disorders and immune dysfunction, thereby posing considerable risks to human and animal health [5,6].

The intestine represents the primary interface between ingested MPs and the host organism. In addition to its essential role in nutrient digestion and absorption, the intestinal tract forms a critical physical, chemical, and immunological barrier that restricts the translocation of harmful microorganisms and toxic substances and contributes to the maintenance of systemic homeostasis [7]. A study has shown that polyethylene microplastics damage the intestinal mucosal structure, increase intestinal permeability, and induce intestinal microbiota dysbiosis and local inflammatory responses, ultimately leading to impaired intestinal barrier function [8]. A damaged intestinal barrier can allow bacterial endotoxins and inflammatory factors to enter the circulatory system, triggering systemic chronic inflammation and affecting the homeostasis of the testicular microenvironment [9]. Increased intestinal permeability and sustained inflammation may disturb testicular homeostasis, compromise blood–testis barrier integrity, and adversely affect spermatogenesis and steroidogenesis [10]. Consistently, experimental exposure to PE-MPs has been associated with impaired spermatogenesis, decreased testosterone concentrations, disruption of endocrine homeostasis, and histopathological alterations in testicular tissue [11,12]. These findings indicate that intestinal barrier dysfunction may represent an important component of PE-MP-induced male reproductive toxicity. However, effective nutritional or naturally derived interventions capable of simultaneously alleviating intestinal and testicular injury following MP exposure remain limited.

Natural polysaccharides have received considerable attention because of their favorable biocompatibility and diverse biological activities, including antioxidant, anti-inflammatory, immunomodulatory, and gut-microbiota-regulating effects. These properties suggest that natural polysaccharides may have considerable potential for mitigating the adverse effects of environmental contaminants [13]. *Artemisia sphaerocephala* Krasch polysaccharide (ASKP), a natural heteropolysaccharide extracted from the seeds of *Artemisia sphaerocephala* Krasch, has been reported to possess remarkable antioxidant activity, immunomodulatory activities and intestinal protective effects [14]. A previous study demonstrated that ASKP enhances antioxidant enzyme activity and average daily weight gain in rats [15], as well as promotes the proliferation of beneficial bacteria such as *Lactobacillus* and *Bifidobacterium* in obese mice [16]. Wu et al. [17] reported that *Cordyceps cicadae* polysaccharides could improve the sperm quality and spermatogenic function of male mice with reproductive damage, alleviate the histological damage of the testes and kidneys, and increase the levels of follicle-stimulating hormone, luteinizing hormone and testosterone. Nevertheless, whether ASKP protects against PE-MP-induced intestinal barrier dysfunction and testicular injury, as well as the underlying mechanisms, remains unclear. Therefore, the present study aimed to investigate the protective effects of crude ASKP preparation against PE-MP-induced intestinal barrier dysfunction and testicular injury in male mice and to further elucidate the potential underlying mechanisms. This study sought to provide experimental evidence for the potential application of crude ASKP preparation as a natural nutritional strategy.

## 2. Materials and Methods

### 2.1. Materials

Male ICR mice were purchased from SPF (Beijing) Biotechnology Co., Ltd. (Beijing, China). The PE-MPs with a nominal diameter of 5 μm were bought from Bomei Biotechnology Co., Ltd. (Hefei, China). The particles were supplied as a powder and were suspended in sterile distilled water by ultrasonication for 30 min before use. The *Artemisia sphaerocephala* seeds were collected in August 2025 from within Ordos City, Inner Mongolia Autonomous Region. Their botanical identity was confirmed by Professor Haizhou Sun. According to the method of Zeng et al. [16], crude ASKP was extracted from *Artemisia sphaerocephala* Krasch seeds by the water extraction and ethanol precipitation method in our laboratory. The crude polysaccharide content was greater than 61.28%. It should be noted that this extraction method may co-precipitate small amounts of water-soluble proteins, polyphenols and oligosaccharides, so the sample used in this study is a crude polysaccharide preparation rather than a chemically pure polysaccharide. The anhydrous ethanol (Purity ≥ 99.7%) was purchased from Tianjin Yongda Chemical Reagent Co., Ltd. (Tianjin, China).

### 2.2. Animal Experiment Design

All animal experiments were approved by the Institutional Animal Care and Use Committee of the Inner Mongolia Academy of Agricultural Sciences (protocol code: 2026-0062). A total of 60 6-week-old healthy male ICR mice with similar body weights (30.15 ± 4.49 g) were randomly divided into 5 groups after 7 days of acclimation: (1) untreated mice (Control group, CON group); (2) mice given oral PE-MPs only (MP group); (3) mice given oral PE-MPs and ASKP (150 mg/kg BW, LAM group); (4) mice given oral PE-MPs and ASKP (300 mg/kg BW, MAM group); and (5) mice given oral PE-MPs and ASKP (450 mg/kg BW, HAM group). Each group of mice were housed in 6 cages, with 2 mice in each cage. Except for the CON group, the other groups were orally administered 0.1 mg/0.2 mL of PE-MPs (5 μm) using a disposable straight feeding needle (20 gauge, 30 mm length) every day throughout the entire experiment, while the CON group was orally given an equal volume of distilled water [18]. According to the dosage studied by Li et al. [19], the LAM, MAM and HAM groups were administered different doses of the crude ASKP preparation every day, while the CON and MP groups were given the equal volume of distilled water. The ASKP and PE-MPs intervention lasted for 60 days. The mice were housed under a 12 h light–dark cycle at a constant temperature of (22 ± 2 °C).

### 2.3. Assessment of Growth Performance

The weights of the mice were measured and recorded on the 1st and 60th days of the formal period, and the average daily weight gain was calculated.

### 2.4. Sample Collection

After the experiment, one mouse from each cage was randomly selected, and fecal samples from the mice were collected and stored at −80 °C. Following anesthesia, blood was rapidly collected from the eyeball and centrifuged to isolate serum, which was stored at −80 °C. Subsequently, the kidneys, spleen, liver and testes were harvested and weighed, and the organ indices were calculated. The right testis and a segment of the jejunum were fixed in 4% paraformaldehyde, and the left testis and portions of jejunum tissue were preserved at −80 °C. The remaining 6 mice per group were also euthanized humanely at the end of the study according to the approved animal protocol.

### 2.5. Jejunal and Testicular Morphology

Intestinal segments and testes were fixed with 4% paraformaldehyde, followed by paraffin embedding, sectioning, hematoxylin and eosin staining, and coverslip mounting. Images were acquired using an Eclipse Ci-L upright brightfield microscope (Nikon Corporation, Tokyo, Japan). The villus height from the tip to the base, crypt depth from the base to mucosa, and villus–crypt ratio were measured.

### 2.6. Biochemical Analysis

The testosterone (ml001948), luteinizing hormone (ml001984) and follicle-stimulating hormone (ml001910) in the serum and the interleukin (IL)-1β (ml106733), IL-6 (ml063159), IL-10 (ml037873), IL-17 (ml037866), tumor necrosis factor (TNF)-α (ml002095) and interferon (IFN)-γ (ml002277) in the jejunum and testes were determined using ELISA kits (Shanghai Enzyme-linked Biotechnology Co., Ltd., Shanghai, China). The diamine oxidase (ADS-F-QT043) and *D*-lactic acid (ADS-F-T017) in the serum and the glutathione peroxidase (GSH-Px, ADS-W-G003), catalase (CAT, ADS-W-KY002), total antioxidant capacity (T-AOC, ADS-W-KY005), superoxide dismutase (SOD, ADS-W-KY011) and malondialdehyde (MDA, ADS-W-YH002) in the jejunum and testes were determined using ELISA kits (Jiangsu Aidisheng Biological Technology Co., Ltd., Yancheng, China). Briefly, approximately 100 mg of jejunal or testicular tissue was homogenized in 1 mL of ice-cold phosphate-buffered saline (PBS, pH 7.4) using a tissue homogenizer (Teledyne Technologies Incorporated, Cincinnati, OH, USA). The homogenate was centrifuged at 12,000 rpm for 10 min at 4 °C, and the supernatant was collected for subsequent assays. The total protein concentration in the supernatant was determined using a BCA protein detection kit (PC0020, Solarbio, Beijing, China), and all biochemical parameters were normalized to the total protein content of the tissue supernatant. All ELISA kits were used according to the manufacturers’ instructions. Each sample was measured in triplicate, and the mean value was used for statistical analysis. The assay units are as follows: IL-6, IL-10, IL-17, TNF-α, and IFN-γ are expressed as pg/mg protein; IL-1β is expressed as ng/mg protein; GSH-Px, CAT, and SOD are expressed as U/g protein; T-AOC and MDA are expressed as μmol Trolox/g and nmol/g protein; serum testosterone, LH, and FSH are expressed as pg/mL, U/L, and nmol/L, respectively; and serum diamine oxidase and D-lactic acid are expressed as nmol/mL and μmol/mL, respectively. All kits used have been widely validated for mouse tissue samples in previous studies, and the detection ranges were confirmed to cover the expected concentrations in mouse jejunum and testis based on preliminary tests and the published literature.

### 2.7. Fecal Microbiota Analysis

The genomic DNA of the feces microbiota was extracted by the Fecal genomic DNA extraction kit (Tiangen Biotech Co., Ltd., Beijing, China). The primer pair 341F/805R was employed to amplify the V3 to V4 regions of the 16S rRNA gene. The DNA libraries were sequenced on the Illumina NovaSeq6000 platform (Illumina Inc., San Diego, CA, USA). Quality filtering of the original tags was performed using QIIME (version 1.2.1). Operational taxonomic units clustering was carried out according to the 97% sequence similarity principle using Uparse software (version 7.0.1001). Linear discriminant analysis effect size (LEfSe) (LDA score = 3) identified the important microbial taxa. Functional genes of the intestinal microbiota were predicted based on a 16S rRNA gene profiling and the database of phylogenetically referenced genomes (PICRUSt). Data on all sequences have been submitted to NCBI under BioProject ID PRJNA1483084.

### 2.8. Statistical Analysis

Statistical analyses were conducted using SPSS Statistics 26.0 (IBM Corp., Chicago, IL, USA). The individual mouse was the experimental unit. The normality of data distribution was tested using the Shapiro–Wilk test, and the homogeneity of variance was verified using Levene’s test. Differences among the five groups were evaluated using Duncan’s multiple range test following one-way ANOVA. For gut microbiota beta diversity analysis, principal component analysis (PCoA) based on unweighted UniFrac distances was performed using QIIME version 1.9.1. Linear discriminant analysis effect size (LEfSe) was used to identify differentially abundant microbial taxa among groups, with a threshold of LDA score > 3. For PICRUSt2-predicted metabolic pathways, differences among groups were analyzed using Welch’s *t*-test, as implemented in the PICRUSt2 pipeline. Spearman’s correlation analysis was performed to assess the associations between biochemical parameters and gut microbiota. Data are presented as mean values, and statistical significance is defined as *p* < 0.05.

## 3. Results

### 3.1. Growth Performance and Organs Indices

**Figure 1:**
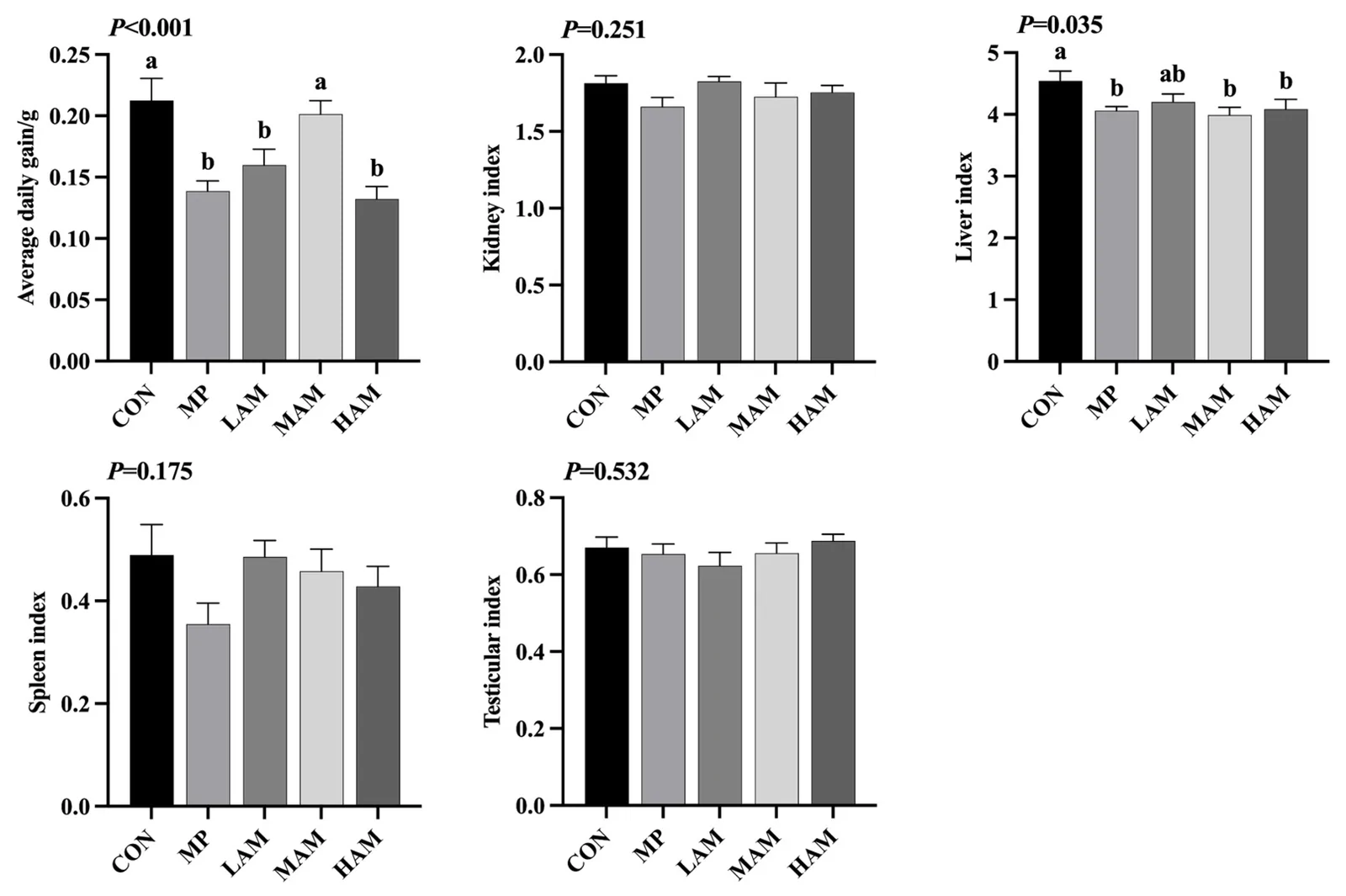
The average daily gain and the organs index among the different groups. CON, basal diet; MP, basal diet + polyethylene-microplastics-induced; LAM, basal diet + 150 mg/kg *Artemisia sphaerocephala* Krasch polysaccharide + polyethylene-microplastics-induced; MAM, basal diet + 300 mg/kg *Artemisia sphaerocephala* Krasch polysaccharide + polyethylene-microplastics-induced; HAM, basal diet + 450 mg/kg *Artemisia sphaerocephala* Krasch polysaccharide + polyethylene-microplastics-induced. ab Different letters indicate significant differences, *p* < 0.05. (*n* = 6 mice).

Compared with the CON group, PE-MPs exposure decreased the average daily gain (Figure 1; *p* < 0.05). Compared with the MP group, the MAM group had a higher average daily gain (*p* < 0.05). The liver index was lower in the MP group than the CON group (*p* < 0.05), while there were no significant differences between the CON and LAM groups.

### 3.2. Intestinal Morphology, Serum Diamine Oxidase and D-Lactic Acid

**Figure 2:**
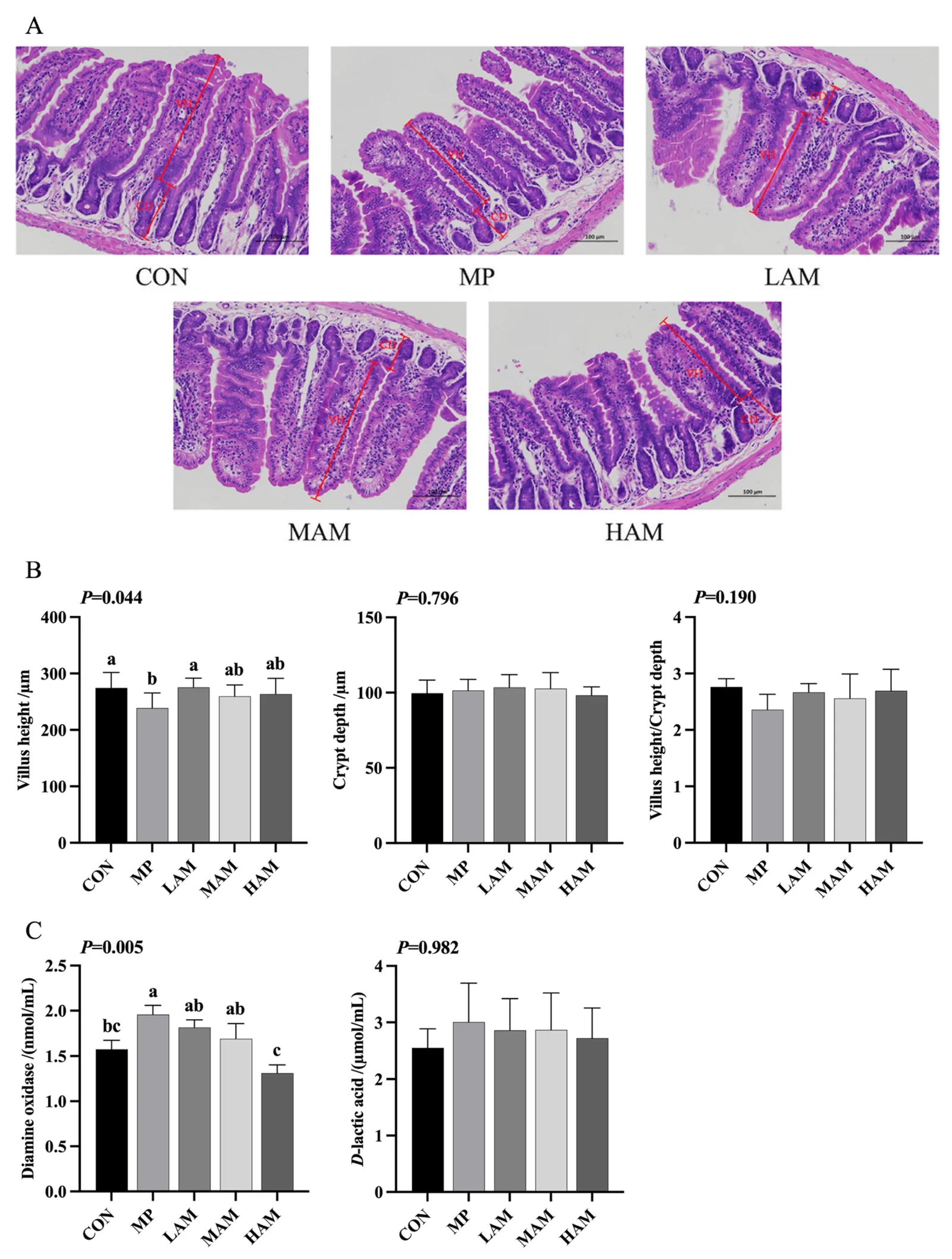
The jejunal morphology (**A**,**B**) and the levels of serum diamine oxidase and *D*-lactic acid (**C**) among the different groups. VH, villus height; CD, crypt depth; CON, basal diet; MP, basal diet + polyethylene-microplastics-induced; LAM, basal diet + 150 mg/kg *Artemisia sphaerocephala* Krasch polysaccharide + polyethylene-microplastics-induced; MAM, basal diet + 300 mg/kg *Artemisia sphaerocephala* Krasch polysaccharide + polyethylene-microplastics-induced; HAM, basal diet + 450 mg/kg *Artemisia sphaerocephala* Krasch polysaccharide + polyethylene-microplastics-induced. abc Different letters indicate significant differences, *p* < 0.05. (*n* = 6 mice).

The jejunal villus height in the CON and LAM groups was higher than that in the MP group (*p* < 0.05), while there were no significant differences among CON, LAM, MAM, and HAM groups (Figure 2A,B). The diamine oxidase level in the CON and HAM groups was lower than that in the MP group (*p* < 0.05), while there were no significant differences among the CON, LAM and MAM groups (Figure 2C).

### 3.3. Jejunal Antioxidant Capacity and Inflammatory Cytokine Levels

**Figure 3:**
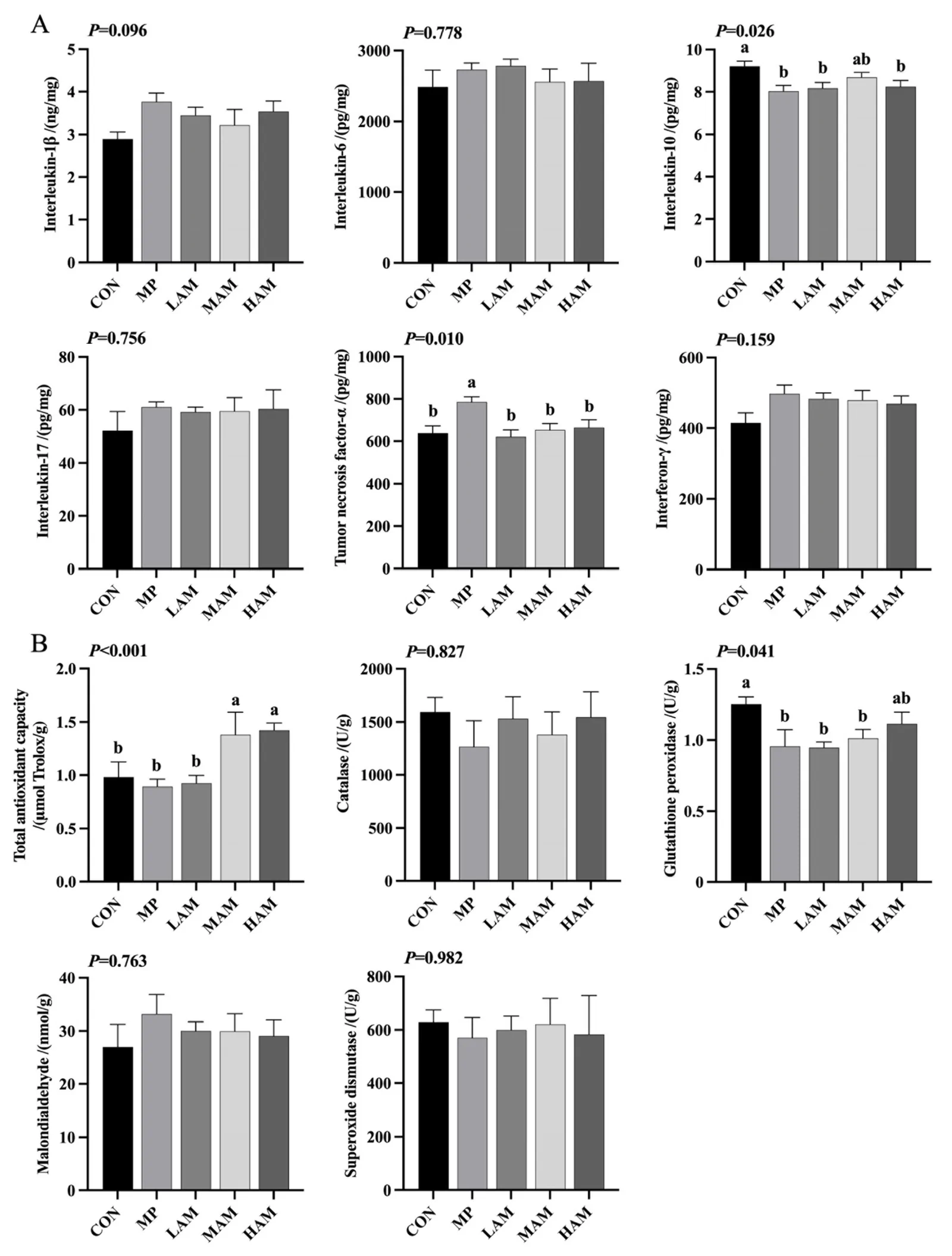
The jejunal inflammatory factor levels (**A**) and antioxidant function (**B**) among the different groups. CON, basal diet; MP, basal diet + polyethylene-microplastics-induced; LAM, basal diet + 150 mg/kg *Artemisia sphaerocephala* Krasch polysaccharide + polyethylene-microplastics-induced; MAM, basal diet + 300 mg/kg *Artemisia sphaerocephala* Krasch polysaccharide + polyethylene-microplastics-induced; HAM, basal diet + 450 mg/kg *Artemisia sphaerocephala* Krasch polysaccharide + polyethylene-microplastics-induced. ab Different letters indicate significant differences, *p* < 0.05. (*n* = 6 mice).

The T-AOC level was significantly higher in the MAM and HAM groups compared with the MP group (Figure 3A,B; *p* < 0.05). The GSH-Px level was lower in the MP group than in the CON group (*p* < 0.05), while there were no significant differences between the CON and HAM groups. The TNF-α level was lower in the CON, LAM, MAM and HAM groups than in the MP group (*p* < 0.05). The IL-10 level was lower in the MP group than in the CON group (*p* < 0.05), while there were no significant differences between CON and MAM groups.

### 3.4. Serum Sex Hormone Levels

**Figure 4:**
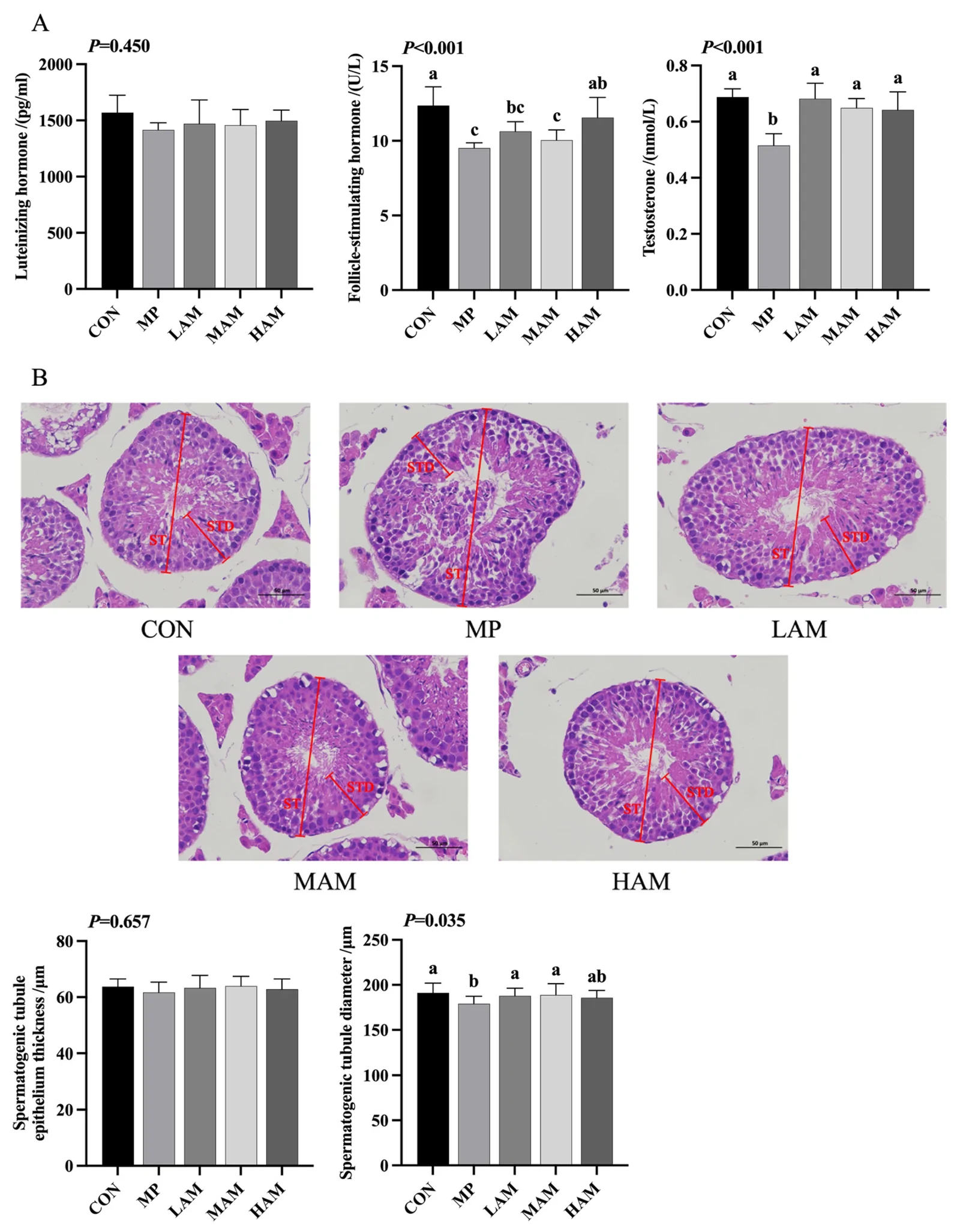
Serum sex hormone levels (**A**) and testicular morphology (**B**) among the different groups. ST, spermatogenic tubule epithelium thickness; STD, spermatogenic tubule diameter; CON, basal diet; MP, basal diet + polyethylene-microplastics-induced; LAM, basal diet + 150 mg/kg *Artemisia sphaerocephala* Krasch polysaccharide + polyethylene-microplastics-induced; MAM, basal diet + 300 mg/kg *Artemisia sphaerocephala* Krasch polysaccharide + polyethylene-microplastics-induced; HAM, basal diet + 450 mg/kg *Artemisia sphaerocephala* Krasch polysaccharide + polyethylene-microplastics-induced. abc Different letters indicate significant differences, *p* < 0.05. (*n* = 6 mice).

The testosterone level in the MP group was lower than those in the other groups (Figure 4A; *p* < 0.05). The follicle-stimulating hormone levels in the CON and HAM groups were higher than that in the MP group (*p* < 0.05).

### 3.5. Testicular Morphology

The lumen morphology of the seminiferous tubules was slightly irregular, the cell arrangement was slightly loose, and the distribution of the sperm bundles in the lumen center was uneven (Figure 4B). Compared with the MP group, the testicular tissue damage in the LAM, MAM and HAM groups improved to varying degrees. The spermatogenic tubule diameters in the CON, LAM and MAM groups were greater than in the MP group (*p* < 0.05), while there were no significant differences between the CON and HAM groups.

### 3.6. Testicular Antioxidant Function and Inflammatory Factor Levels

**Figure 5:**
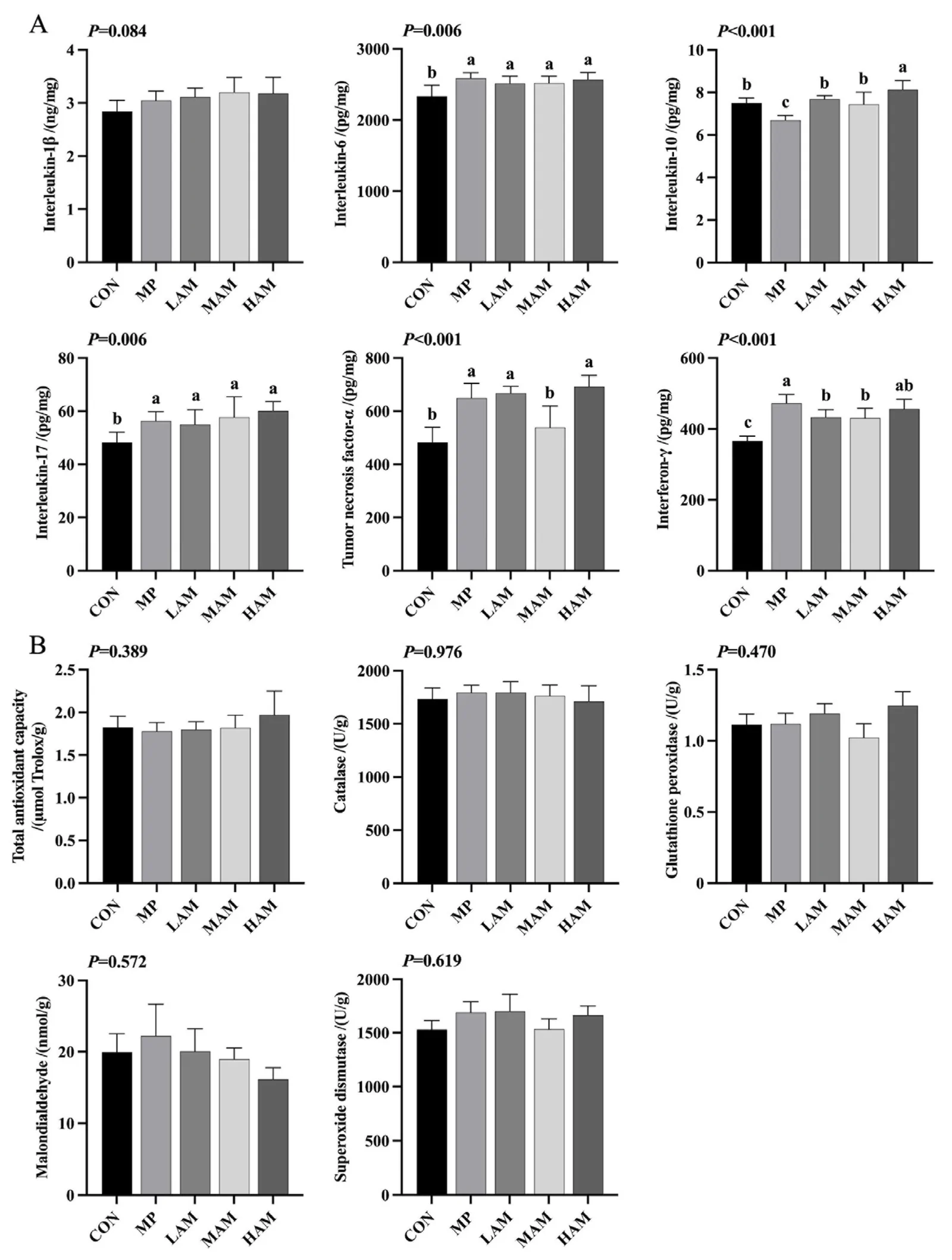
The testicular inflammatory factor levels (**A**) and antioxidant function (**B**) among the different groups. CON, basal diet; MP, basal diet + polyethylene-microplastics-induced; LAM, basal diet + 150 mg/kg *Artemisia sphaerocephala* Krasch polysaccharide + polyethylene-microplastics-induced; MAM, basal diet + 300 mg/kg *Artemisia sphaerocephala* Krasch polysaccharide + polyethylene-microplastics-induced; HAM, basal diet + 450 mg/kg *Artemisia sphaerocephala* Krasch polysaccharide + polyethylene-microplastics-induced. abc Different letters indicate significant differences, *p* < 0.05. (*n* = 6 mice).

The IL-6, IL-17, TNF-α and IFN-γ levels increased (*p* < 0.05), while the IL-10 level decreased in the MP group compared with the CON group (*p* < 0.05) (Figure 5A,B). Compared with the MP group, the IL-10 level increased in the LAM, MAM and HAM groups (*p* < 0.05), and the TNF-α level decreased in the MAM group (*p* < 0.05), while the IFN-γ level decreased in the LAM and MAM groups (*p* < 0.05).

### 3.7. The α-Diversity and β-Diversity Indices of the Fecal Microbiota

**Figure 6:**
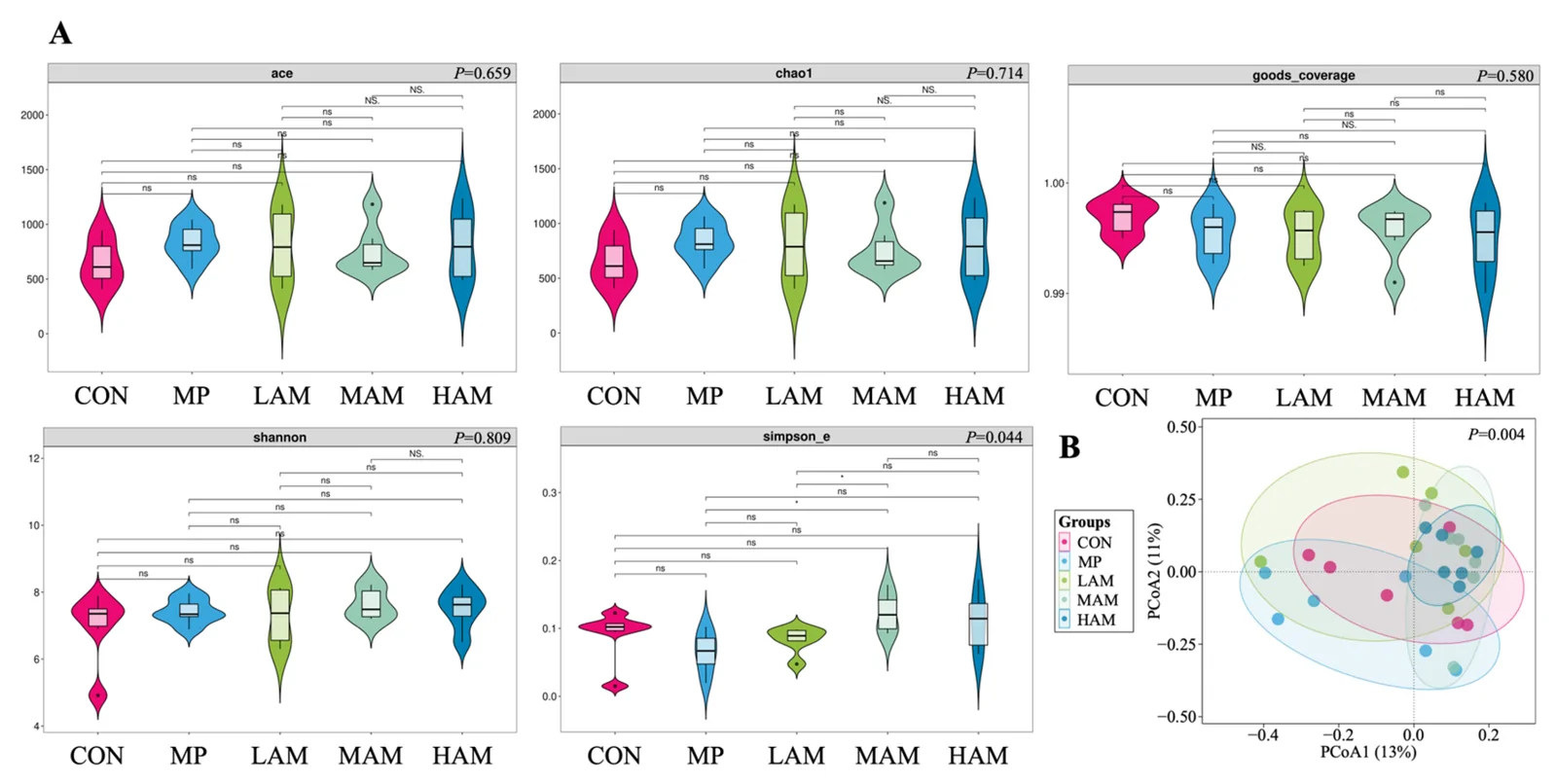
The α diversity (**A**) and β diversity (**B**) of the fecal microbiota among the different groups. CON, basal diet; MP, basal diet + polyethylene-microplastics-induced; LAM, basal diet + 150 mg/kg *Artemisia sphaerocephala* Krasch polysaccharide + polyethylene-microplastics-induced; MAM, basal diet + 300 mg/kg *Artemisia sphaerocephala* Krasch polysaccharide + polyethylene-microplastics-induced; HAM, basal diet + 450 mg/kg *Artemisia sphaerocephala* Krasch polysaccharide + polyethylene-microplastics-induced. *, significant differences, *p* < 0.05; ns or NS., not significant, *p* ≥ 0.05. (*n* = 6 mice).

The simpson_e index in the MAM group was higher than in the MP and LAM groups (Figure 6A,B; *p* < 0.05). The groups differed significantly in the β-diversity index (*p* < 0.05).

### 3.8. Abundance of Fecal Microbiota

**Figure 7:**
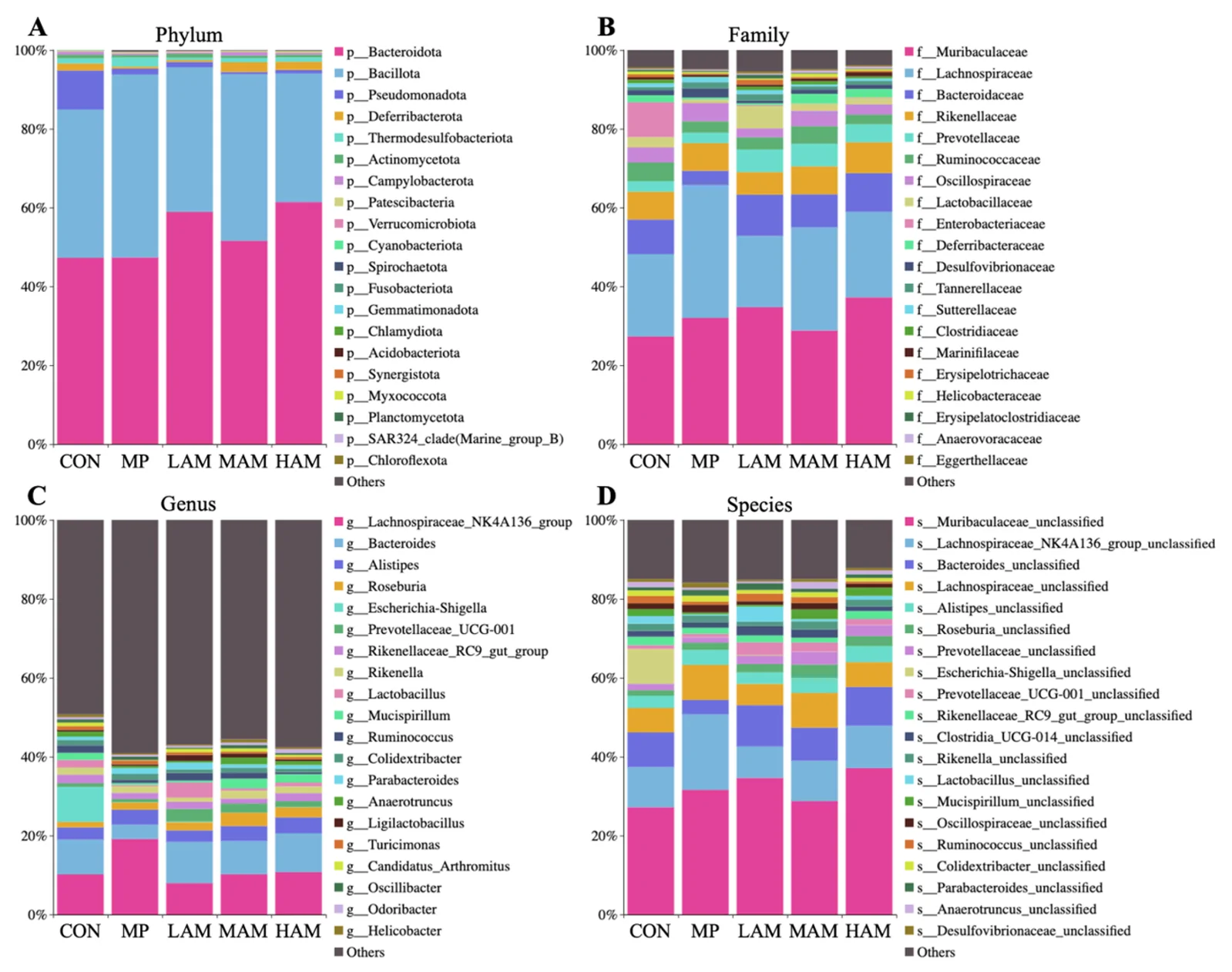
The relative abundance of the fecal microbiota at the phylum (**A**), family (**B**), genus (**C**), and species (**D**) levels among the different groups. CON, basal diet; MP, basal diet + polyethylene-microplastics-induced; LAM, basal diet + 150 mg/kg *Artemisia sphaerocephala* Krasch polysaccharide + polyethylene-microplastics-induced; MAM, basal diet + 300 mg/kg *Artemisia sphaerocephala* Krasch polysaccharide + polyethylene-microplastics-induced; HAM, basal diet + 450 mg/kg *Artemisia sphaerocephala* Krasch polysaccharide + polyethylene-microplastics-induced. (*n* = 6 mice).

Compared with the CON group, PE-MPs exposure decreased the abundances of p__Deferribacterota, f__Enterobacteriaceae, f__Eggerthellaceae, f__Clostridiaceae, f__Deferribacteraceae, g_*_Adlercreutzia*, g__*Mucispirillum*, g_*_Escherichia-Shigella* and g_*_Candidatus Arthromitus* (*p* < 0.05) and increased the abundances of f__Bacillaceae and s__*Bacillus_cereus* in the feces (Figure 7 and Tables S1–S4; *p* < 0.05). Compared with the MP group, the LAM group showed significantly decreased abundances of p__Thermodesulfobacteriota, p__Patescibacteria, f__Saccharimonadaceae, f__Oscillospiraceae, g__*Acutalibacter*, f__Butyricicoccaceae, f__Desulfovibrionaceae, g_*_Tyzzerella*, g_*_Desulfovibrio*, g_*_Candidatus Saccharimonas*, g_*_Colidextribacter* and g_*_Oscillibacter* (*p* < 0.05) and showed significantly increased abundances of f__Bacteroidaceae, f__Clostridiaceae, g_*_Prevotellaceae UCG-001*, g*__Candidatus Arthromitus* and g*__Bacteroides* (*p* < 0.05). The MAM group showed significantly decreased abundances of p__Thermodesulfobacteriota, f__Bacillaceae, f__Desulfovibrionaceae and s_*_Bacillus_cereus* and showed significantly increased abundances of p__Deferribacterota, f__Deferribacteraceae, f__Anaerovoracaceae, f__Bacteroidaceae, f__Clostridiaceae, g_*_Anaerotruncus*, g__*Mucispirillum*, g_*_Bacteroides* and g_*_Candidatus Arthromitus* (*p* < 0.05). The HAM group showed significantly decreased abundance of f__Butyricicoccaceae (*p* < 0.05) and showed significantly increased abundances of f__Christensenellaceae, f__Anaerovoracaceae, g__*Candidatus Arthromitus*, f__Clostridiaceae and g*__Prevotellaceae UCG-001* (*p* < 0.05).

### 3.9. LEfSe Analysis

**Figure 8:**
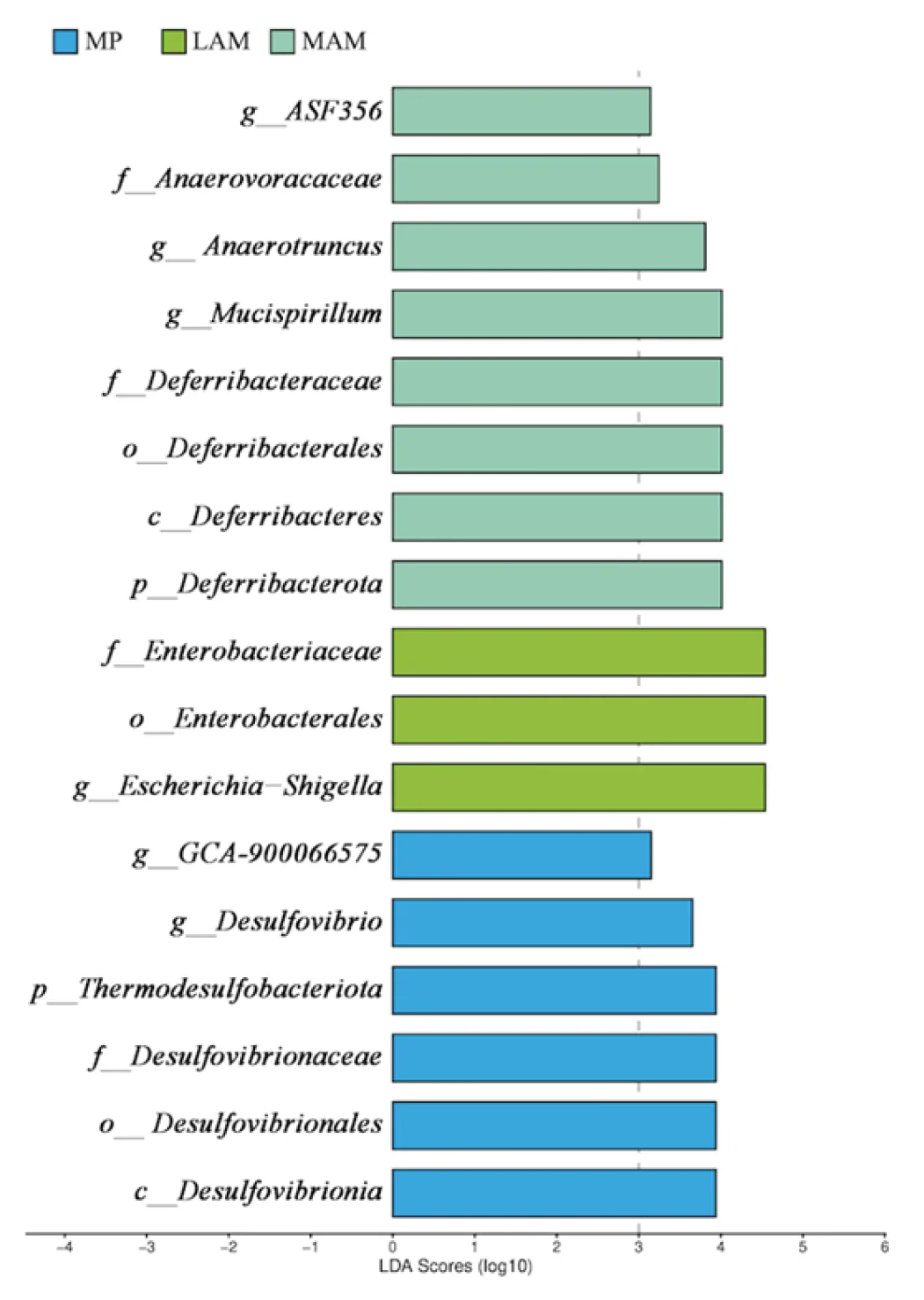
The LEfSe of the fecal microbiota among the different groups. MP, basal diet + polyethylene-microplastics-induced; LAM, basal diet + 150 mg/kg *Artemisia sphaerocephala* Krasch polysaccharide + polyethylene-microplastics-induced; MAM, basal diet + 300 mg/kg *Artemisia sphaerocephala* Krasch polysaccharide + polyethylene-microplastics-induced. (*n* = 6 mice).

Taxa that were significantly differentially represented among the groups were examined by LEfSe (Figure 8; LDA score  =  3). p__Thermodesulfobacteriota, c__Desulfovibrionia, o__Desulfovibrionales, f__Desulfovibrionaceae, *g*__*GCA-900066575* and *g*__*Desulfovibrio* were abundant in the MP group. o__Enterobacterales, f__Enterobacteriaceae and g__*Escherichia-Shigella* were abundant in the LAM group. p__Deferribacterota, c__Deferribacteres, o__Deferribacterales, f__Anaerovoracaceae, f__Deferribacteraceae, g__*Anaerotruncus*, g__*Mucispirillum* and g__*ASF356* were abundant in the MAM group.

### 3.10. Functional Prediction of Gut Microbiota

**Figure 9:**
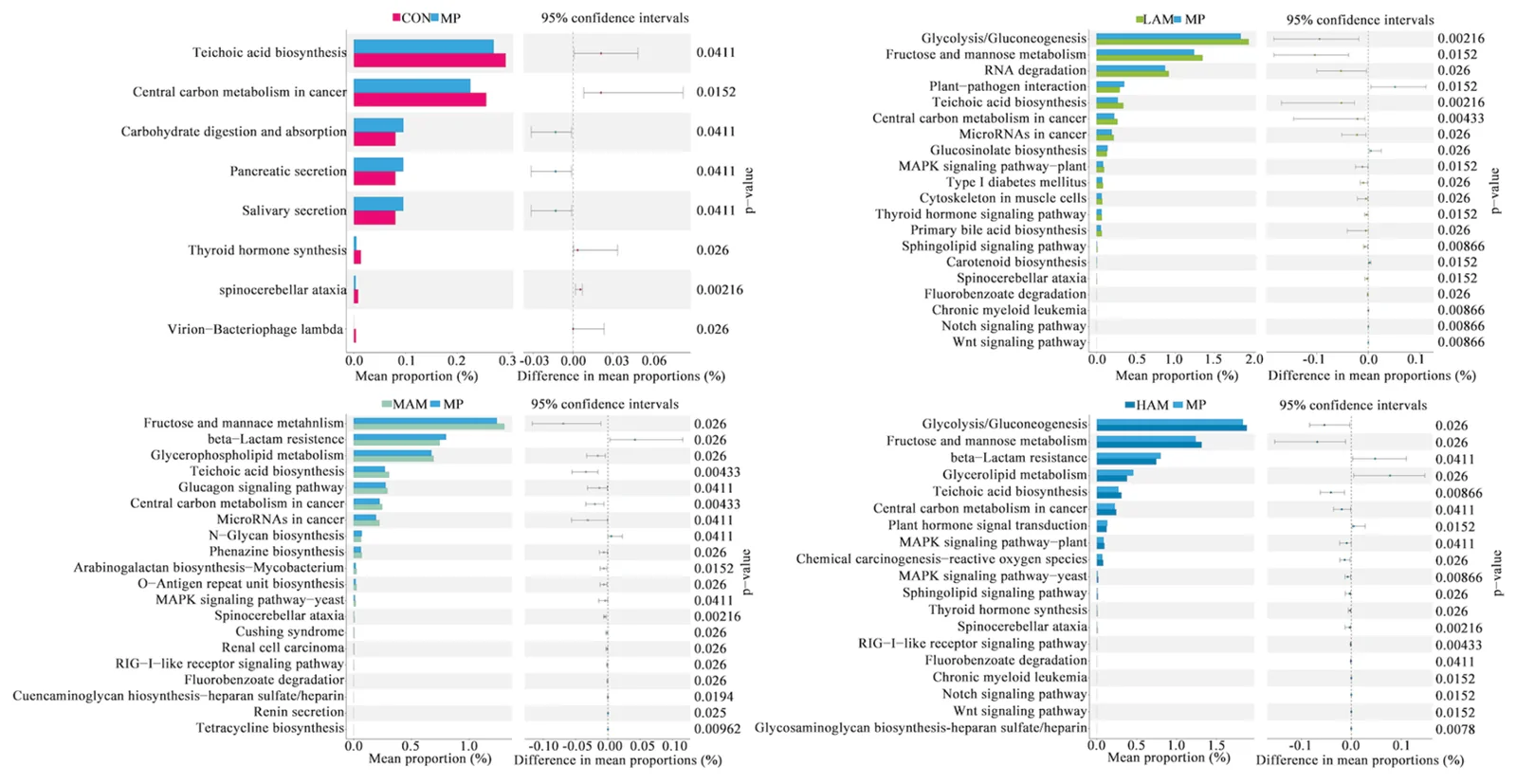
The function prediction of the fecal microbiota among the different groups. CON, basal diet; MP, basal diet + polyethylene-microplastics-induced; LAM, basal diet + 150 mg/kg *Artemisia sphaerocephala* Krasch polysaccharide + polyethylene-microplastics-induced; MAM, basal diet + 300 mg/kg *Artemisia sphaerocephala* Krasch polysaccharide + polyethylene-microplastics-induced; HAM, basal diet + 450 mg/kg *Artemisia sphaerocephala* Krasch polysaccharide + polyethylene-microplastics-induced. (*n* = 6 mice).

Compared with the CON group, the MP group downregulated teichoic acid biosynthesis, thyroid hormone synthesis, and virion–bacteriophage lambda, while it upregulated carbohydrate digestion and absorption, pancreatic secretion, and salivary secretion (Figure 9). Compared with the MP group, the LAM group downregulated plant–pathogen interaction, glucosinolate biosynthesis, and carotenoid biosynthesis, while it upregulated glycolysis/gluconeogenesis, fructose and mannose metabolism, RNA degradation, teichoic acid biosynthesis, etc. The MAM group downregulated beta-lactam resistance and *N*-glycan biosynthesis, while it upregulated fructose and mannose metabolism, glycerophospholipid metabolism, teichoic acid biosynthesis, glucagon signaling pathway, phenazine biosynthesis, arabinogalactan biosynthesis–mycobacterium, O-antigen repeat unit biosynthesis, etc. The HAM group downregulated beta-Lactam resistance, glycerolipid metabolism, and plant hormone signal transduction, and it upregulated the glycolysis/gluconeogenesis, fructose and mannose metabolism, teichoic acid biosynthesis, chemical carcinogenesis–reactive oxygen species, sphingolipid signaling pathway, etc.

### 3.11. Spearman Correlation Analyses

**Figure 10:**
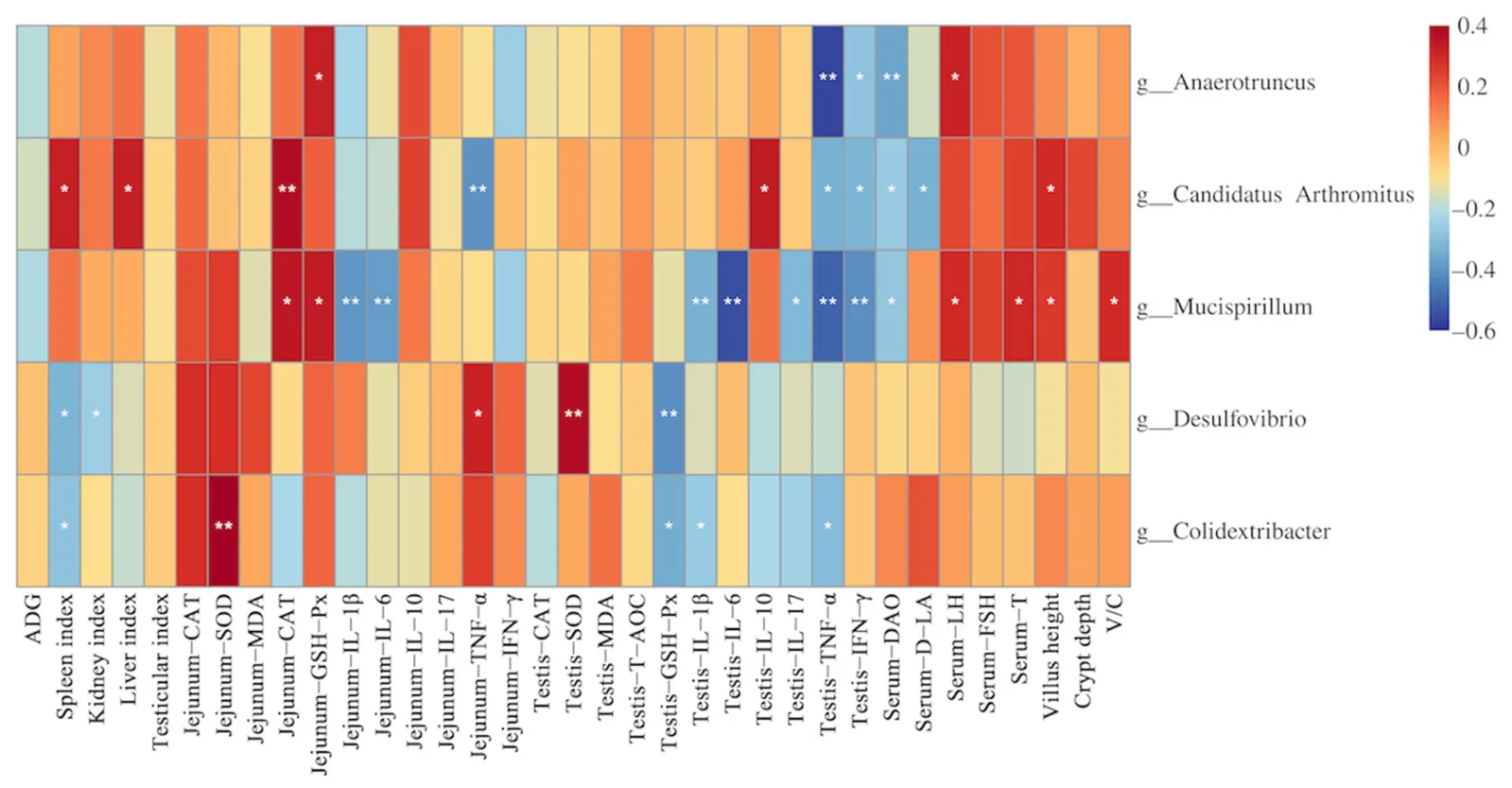
The spearman correlation analyses between the biochemical parameters and gut microbiota. CON, basal diet; MP, basal diet + polyethylene-microplastics-induced; LAM, basal diet + 150 mg/kg *Artemisia sphaerocephala* Krasch polysaccharide + polyethylene-microplastics-induced; MAM, basal diet + 300 mg/kg *Artemisia sphaerocephala* Krasch polysaccharide + polyethylene-microplastics-induced; HAM, basal diet + 450 mg/kg *Artemisia sphaerocephala* Krasch polysaccharide + polyethylene-microplastics-induced. (*n* = 6 mice). *, *p* < 0.05; **, *p* < 0.01.

A correlation analysis was conducted on the factors with correlation coefficients (r) that were greater than 0.4 or less than −0.4 (Figure 10). The abundance of g_*_Mucispirillum* was negatively correlated with the testicular IL-6 level (r = −0.54), TNF-α level (r = −0.50) and IFN-γ level (r = −0.41) (*p* < 0.05). The abundance of g_*_Anaerotruncus* was negatively correlated with the testicular TNF-α level (r = −0.56) (*p* < 0.05). The abundance of g_*_Colidextribacter* was positively correlated with the jejunal SOD level (r = 0.42) (*p* < 0.05). The abundance of g_*_Desulfovibrio* was negatively correlated with the testicular GSH-Px level (r = −0.41) (*p* < 0.05).

## 4. Discussion

Exposure to PE-MPs resulted in a significant decrease in the average daily gain in mice, indicating the negative impact of MPs on the overall nutritional metabolism and health status of the organism. This result is consistent with the finding reported by a study that PE-MPs can inhibit weight gain by mice [3]. However, supplementation with 300 mg/kg crude ASKP preparation can alleviate the decline in production performance caused by PE-MPs. The high-dose crude ASKP preparation did not further enhance growth performance. This may be because high doses of polysaccharides alter the abundance of various intestinal bacteria, trigger intestinal inflammation, and thereby fail to exert growth-promoting effects [20]. However, feed intake was not reported in this study, and these possibilities cannot be distinguished using the available data. Further research is needed in the future to explore this. The organ index can directly reflect the impact of exogenous pollutants on the development and degree of damage to the organs. Yan et al. [21] found that *Lycium barbarum* polysaccharides could increase the kidney weight and organ index of mallard ducks induced by 10 mg/kg polyvinyl chloride microplastics. In this study, the liver index in the MP group decreased significantly, and it recovered after crude ASKP preparation intervention. The liver, as the main detoxification and metabolism center in the body, experiences a relative weight reduction, which may indicate liver cell damage induced by PE-MPs [22].

The intestinal epithelial barrier serves as the first line of defense against exogenous pollutants and the invasion of intestinal symbiotic bacteria [23]. This study showed that exposure to PE-MPs significantly reduces the height of the intestinal villi in mice, accompanied by villus atrophy and impaired epithelial integrity. This indicates that PE-MPs can directly cause frictional damage to intestinal villus epithelial cells, thereby destroying the structure of the small intestinal epithelium. Diamine oxidase, as an intracellular enzyme in small intestinal mucosal epithelial cells, is released into the bloodstream in large quantities when epithelial cells are damaged. In this study, exposure to PE-MPs increased the serum diamine oxidase level [24]. This was consistent with a study by Xia et al. [25], who also found that PE-MPs cause damage to the intestinal morphology of loaches and an increase in the serum diamine oxidase level. In contrast, crude ASKP preparations can increase the jejunal villus height and reduce the serum diamine oxidase level, indicating that crude ASKP preparations can alleviate the damage caused by PE-MPs to the intestinal barrier. In addition, the disruption of the intestinal barrier function is often accompanied by intestinal inflammation and oxidative stress [26]. In our study, exposure to PE-MPs significantly reduced the activities of T-AOC and GSH-Px in the jejunum, while increasing the level of pro-inflammatory factor TNF-α and decreasing the level of anti-inflammatory factor IL-10, which is consistent with the study by Zeng et al. [27]. However, crude ASKP preparation supplementation can increase T-AOC and GSH-Px levels and reduce TNF-α to the level of the control group. Combining previous antioxidant activity studies on ASKP [14], ASKP can alleviate the intestinal damage induced by PE-MPs by enhancing the activity of antioxidant enzymes in the body and inhibiting the secretion of pro-inflammatory factors. Zhang et al. [28] found that dietary supplementation with 24 mg/kg of polysaccharides from *Opuntia Milpa Alta* reduced SOD and CAT activities, restored GSH and normalized MDA levels with 5 µm of polypropylene microplastics.

We further examined the levels of inflammatory factors and oxidative stress in the testicles. Exposure to PE-MPs did not cause significant changes in antioxidant indicators such as CAT, SOD, and MDA. However, the pro-inflammatory factors IL-6, IL-17, TNF-α, and IFN-γ increased, while the anti-inflammatory factor IL-10 decreased. This suggests that the testicular damage induced by PE-MPs was mainly characterized by an inflammatory response under these experimental conditions. The crude ASKP preparation treatment alleviated the inflammatory state of the testicles. Tang et al. [29] also found that *Epimedium* polysaccharide could reduce the levels of IL-1β, IL-6, and TNF-α in the testes of oligoasthenozoospermia mice. The stability of sex hormones and the integrity of the spermatogenic epithelium are the core foundations for maintaining male reproductive functions [30]. In our study, PE-MPs decreased the testosterone and follicle-stimulating hormone levels in the serum of mice, while the arrangement of spermatogenic cells became loose and the lumen structure became irregular. Yang et al. [31] found that PE-MPs exposure impaired the testicular testosterone synthesis, resulting in decreased sperm motility and lower serum testosterone levels. This indicates that PE-MPs may have reproductive toxicity towards male mice by causing endocrine disorders in reproduction. Our results suggest that the crude ASKP preparation treatment increased the testosterone and follicle-stimulating hormone levels. These findings indicate that ASKP ameliorates PE-MP-induced testicular histological changes and reproductive hormone disturbances in mice, but further studies including sperm quality and fertility assays are required to confirm functional reproductive protection. Zhang et al. [32] suggested that *Lycium barbarum* polysaccharides can increase testosterone and luteinizing hormone levels on testis spermatogenic injury induced by bisphenol A in mice.

The gut microbiota serves as the central hub connecting intestinal health with the functions of distant organs and is also a key regulatory target of the gut–reproductive axis. This study found that PE-MPs exposure changed the β diversity, increased the abundance of opportunistic pathogenic bacteria such as s__*Bacillus cereus*, while decreasing the g_*_Adlercreutzia,* g_*_Mucispirillum* and g_*_Candidatus Arthromitus* abundances. A study has shown that PE-MPs decreased the abundance of the probiotics *Mucispirillum* in the intestines of mice [33]. We showed that crude ASKP preparations can significantly enhance the α diversity of the microbiota, increase the abundances of g*__Candidatus Arthromitus,* g_*_Mucispirillum* and g_*_Anaerotruncus*, and simultaneously reduce g*__Desulfovibrio,* g*__Colidextribacter* and s_*_Bacillus cereus* abundances. Studies have found that polysaccharides can increase g*__Candidatus Arthromitus* abundance in constipated mice [34] and g_*_Mucispirillum* and g*__Anaerotruncus* abundances in mice with ulcerative colitis [35,36]. *Candidatus Arthromitus* is a well-documented symbiont that promotes intestinal immune maturation and helps to establish immune homeostasis [37], while *Anaerotruncus,* as a type of butyric-acid-producing bacteria, plays a crucial role in maintaining the integrity of the intestinal barrier and in anti-inflammatory processes [38]. This suggests that crude ASKP preparations may be associated with increased production of short-chain fatty acids by bacteria such as butyrate-producing *Anaerotruncus*, which in turn may contribute to intestinal barrier repair and inhibition of inflammatory responses. However, this hypothesis remains to be verified by direct measurement of SCFA concentrations. The results of the functional prediction of the microbiota showed that PE-MPs exposure could alter synthesis of secondary metabolites and inflammatory-related signaling pathways, while crude ASKP preparation intervention could regulate the metabolic pathways related to oxidative stress and inflammation. This further corroborates our findings on oxidative stress, immune indicators, and gut microbiota, suggesting that crude ASKP preparations can influence the physiological state of mice by regulating the metabolic function of the flora.

PE-MPs have been increasingly recognized as disrupting the gut microbial ecosystem, thereby contributing to systemic inflammation and reproductive dysfunction through the gut–reproductive axis. In the present study, correlation analysis further supported the close association between specific gut microbial taxa and inflammatory or oxidative stress markers in the testes and intestine. Our studies found that the g_*_Colidextribacter* exhibited a positive correlation with jejunal SOD activity. Oxidative stress is considered one of the central mechanisms responsible for PE-MP-induced intestinal and reproductive toxicity [39]. Enhanced antioxidant capacity in the intestine may reduce reactive oxygen species production, preserve epithelial barrier integrity, and, consequently, limit the translocation of inflammatory mediators into circulation [40]. Previous microbiome studies have associated *Colidextribacter* with improved metabolic homeostasis and antioxidant status in several disease models [41,42]. Therefore, the positive correlation between *Colidextribacter* abundance and jejunal SOD activity suggests that this bacterium may participate in maintaining intestinal redox balance, indirectly contributing to protection of the reproductive system. In addition, g_*_Anaerotruncus* was negatively correlated with testicular TNF-α levels in our study. Members of this genus belong to short-chain fatty acid-producing anaerobes, and several studies have suggested that short-chain fatty acids, particularly butyrate, exert anti-inflammatory effects by enhancing intestinal barrier function and suppressing nuclear-factor-κB-mediated inflammatory signaling [43,44]. Nevertheless, it should be emphasized that the present results are based on correlation analyses, and further mechanistic studies are required to clarify the causal relationship.

Several limitations of this study should be acknowledged. First, no ASKP-only control group without PE-MP exposure was included, so we cannot distinguish whether the observed effects represent rescue from microplastic damage or independent beneficial effects of ASKP under physiological conditions. Second, the effective sample size was relatively small (*n* = 6 per group), which may limit statistical power for detecting small effect sizes, and multiple testing correction was not applied to microbiota and functional pathway data; thus, some findings should be interpreted as exploratory. Third, gut microbiota was analyzed in fecal samples rather than site-matched jejunal mucosa, and functional predictions were based on 16S rRNA gene sequencing rather than direct measurement of microbial metabolites such as short-chain fatty acids. Fourth, only testicular histology and hormone levels were assessed; sperm quality parameters and fertility outcomes were not measured, so functional reproductive protection cannot be concluded. Fifth, this study was conducted in a single mouse strain and model, and the generalizability to other species or human populations remains to be determined. Future studies with larger sample sizes, factorial designs, multi-omics approaches and functional fertility endpoints are warranted to validate and extend our findings.

## 5. Conclusions

Oral administration of crude ASKP preparation can increase the jejunal villus height, improve intestinal barrier function and antioxidant capacity, and reduce intestinal inflammation in PE-MP-induced mice. The crude ASKP preparation-mediated alterations in intestinal microbiota (g__*Anaerotruncus*, g__*Candidatus Arthromitus*, g__*Desulfovibrio*, and g__*Colidextribacter*) were associated with increased serum follicle-stimulating hormone and testosterone levels, improved testicular morphology, reduced inflammatory factor levels, and improved growth performance. Overall, under the experimental conditions of this study, a dose of 300 mg/kg BW of crude ASKP preparation is preferable.

## Data Availability

The original contributions presented in this study are included in the article/Supplementary Materials. Further inquiries can be directed to the corresponding author.

## Supplementary Materials

The following supporting information can be downloaded at: https://www.mdpi.com/article/10.3390/nu18193224/s1, Table S1: Significantly altered bacteria between the CON and MP groups; Table S2: Significantly altered bacteria between the MP and LAM groups; Table S3: Significantly altered bacteria between the MP and MAM groups; Table S4: Significantly altered bacteria between the MP and HAM groups.

## Abbreviations

The following abbreviations are used in this manuscript:

ASKP	*Artemisia sphaerocephala* Krasch polysaccharide
PE	Polyethylene
PE-MPs	polyethylene microplastics
IL	Interleukin
TNF-α	Tumor necrosis factor α
IFN-γ	Interferon γ
GSH-Px	Glutathione peroxidase
CAT	Catalase
T-AOC	Total antioxidant capacity
SOD	Superoxide dismutase
MDA	Malondialdehyde
