# Supplementary material for: Protective Effects of Artemisia sphaerocephala Krasch Polysaccharide Against Polyethylene-Microplastic-Induced Intestinal and Testicular Injury in Male Mice
Source: Nutrients. 2026 Sep 29;18(19):3224. doi: 10.3390/nu18193224 (PMC13667171; doi:10.3390/nu18193224)
Supplement: Supplementary file 1 [file nutrients-18-03224-s001.zip › nutrients-4553118-supplementary.pdf]

Table S1. Significantly altered bacteria between the CON and MP groups.

Bacteria	up/down	Log2FC	*P*-value
p__Bacteria_unclassified	up	4.28	0.06
p__unclassified	up	2.64	0.07
p__Deferribacterota	down	-1.85	0.19
f__Bacillaceae	up	3.49	0.17
f__Bacteria_unclassified	up	4.28	0.17
f__unclassified	up	2.64	0.23
f__Eggerthellaceae	down	-1.32	0.23
f__Enterobacteriaceae	down	-6.91	0.41
f__Bacteroidales_unclassified	up	3.44	0.41
f__Deferribacteraceae	down	-1.85	0.41
f__Clostridiaceae	down	-2.75	0.41
g__Bacillus	up	3.49	0.35
g__Bacteria_unclassified	up	4.28	0.35
g__unclassified	up	2.64	0.44
g__Adlercreutzia	down	-1.31	0.44
g__UCG-002	down	-3.58	0.44
g__UCG-003	up	3.17	0.48
g__GCA-900066575	up	2.73	0.51
g__Escherichia-Shigella	down	-7.12	0.60
g__Bacteroidales_unclassified	up	3.44	0.60
g__Mucispirillum	down	-1.85	0.60
g__Candidatus_Arthromitus	down	-2.92	0.60
g__Lachnospiraceae_UCG-006	up	1.16	0.61
s__Bacillus_cereus	up	5.07	0.37
s__Bacteria_unclassified	up	4.28	0.37
s__unclassified	up	2.64	0.47
s__Adlercreutzia_unclassified	down	-1.31	0.47
s__UCG-002_unclassified	down	-3.58	0.47
s__UCG-003_unclassified	up	3.17	0.52
s__GCA-900066575_unclassified	up	2.73	0.54
s__Escherichia-Shigella_unclassified	down	-7.12	0.59
s__Bacteroidales_unclassified	up	3.44	0.59
s__Mucispirillum_unclassified	down	-1.85	0.59
s__Candidatus_Arthromitus_unclassified	down	-2.92	0.59
s__Lachnospiraceae_UCG-006_unclassified	up	1.16	0.59

CON, basal diet; MP, basal diet + microplastics challenge.

Table S2. Significantly altered bacteria between the MP and LAM groups.

Bacteria	up/down	Log2FC	*P*-value
p__Thermodesulfobacteriota	down	-1.97	0.19
p__Patescibacteria	down	-0.86	0.22

f__Oscillospiraceae	down	-1.02	0.45
f__Desulfovibrionaceae	down	-1.97	0.45
f__Bacteroidaceae	up	1.53	0.45
f__Enterococcaceae	up	3.75	0.45
f__Saccharimonadaceae	down	-0.87	0.49
f__Butyricicoccaceae	down	-1.25	0.49
f__Clostridiaceae	up	2.70	0.49
f__Mitochondria	up	3.32	0.49
g__Ruminococcaceae_unclassified	down	-1.42	0.49
g__Desulfovibrio	down	-2.58	0.49
g__Acutalibacter	down	-1.11	0.49
g__UCG-009	down	-1.52	0.49
g__Bacteroides	up	1.53	0.49
g__Enterococcus	up	3.75	0.49
g__Oscillospiraceae_unclassified	down	-1.11	0.49
g__Candidatus_Saccharimonas	down	-0.88	0.49
g__Tyzzerella	down	-1.36	0.49
g__UCG-003	down	-2.66	0.49
g__Prevotellaceae_UCG-001	up	1.95	0.49
g__Colidextribacter	down	-0.70	0.49
g__Oscillibacter	down	-1.46	0.49
g__Candidatus_Arthromitus	up	2.88	0.49
g__Mitochondria_unclassified	up	3.32	0.49
s__Ruminococcaceae_unclassified	down	-1.42	0.49
s__Desulfovibrio_unclassified	down	-2.58	0.49
s__Acutalibacter_unclassified	down	-1.11	0.49
s__UCG-009_unclassified	down	-1.52	0.49
s__Bacteroides_unclassified	up	1.53	0.49
s__Enterococcus_unclassified	up	Inf	0.49
s__Oscillospiraceae_unclassified	down	-1.11	0.49
s__Candidatus_Saccharimonas_unclassified	down	-0.88	0.49
s__Tyzzerella_unclassified	down	-1.36	0.49
s__UCG-003_unclassified	down	-2.66	0.49
s__Prevotellaceae_UCG-001_unclassified	up	1.95	0.49
s__Colidextribacter_unclassified	down	-0.70	0.49
s__Oscillibacter_unclassified	down	-1.46	0.49
s__Candidatus_Arthromitus_unclassified	up	2.88	0.49
s__Mitochondria_unclassified	up	3.32	0.49

MP, basal diet + microplastics challenge; LAM, basal diet + 150 mg/kg artemisia deserti polysaccharide + microplastics challenge.

Table S3. Significantly altered bacteria between the MP and MAM groups.

**Bacteria**	**up/down**	**Log2FC**	***P*-value**
p__Bacteria_unclassified	down	-3.28	0.09

p__Thermodesulfobacteriota	down	-1.11	0.13
p__Deferribacterota	up	2.32	0.13
f__Bacteria_unclassified	down	-3.28	0.29
f__Clostridiaceae	up	2.62	0.29
f__Bacteroidales_unclassified	down	-4.02	0.29
f__Corynebacteriaceae	down	-Inf	0.29
f__Anaerovoracaceae	up	1.06	0.29
f__Bacteroidaceae	up	1.21	0.29
f__Desulfovibrionaceae	down	-1.11	0.29
f__Deferribacteraceae	up	2.32	0.29
f__Anaerofustaceae	up	2.81	0.34
f__Bacillaceae	down	-1.41	0.34
f__Flavobacteriaceae	up	2.12	0.34
g__Bacteria_unclassified	down	-3.28	0.39
g__Anaerotruncus	up	1.69	0.39
g__ASF356	up	2.65	0.39
g__Paraprevotella	up	3.31	0.39
g__Candidatus_Arthromitus	up	2.78	0.39
g__UCG-003	down	-3.17	0.39
g__Bacteroidales_unclassified	down	-4.02	0.39
g__Corynebacterium	down	-Inf	0.39
g__Anaerovoracaceae_unclassified	up	1.73	0.39
g__Bacteroides	up	1.21	0.39
g__Mucispirillum	up	2.32	0.39
g__Ruminococcaceae_unclassified	down	-0.78	0.39
g__Anaerofustis	up	2.81	0.48
g__Bacillus	down	-1.41	0.49
g__Family_XIII_AD3011_group	up	1.32	0.51
g__Flavobacteriaceae_unclassified	up	2.12	0.51
s__Bacteria_unclassified	down	-3.28	0.44
s__Anaerotruncus_unclassified	up	1.69	0.44
s__ASF356_unclassified	up	2.65	0.44
s__Paraprevotella_unclassified	up	3.31	0.44
s__Candidatus_Arthromitus_unclassified	up	2.78	0.44
s__UCG-003_unclassified	down	-3.17	0.44
s__Bacteroidales_unclassified	down	-4.02	0.44
s__Anaerovoracaceae_unclassified	up	1.73	0.44
s__Bacteroides_unclassified	up	1.21	0.44
s__Mucispirillum_unclassified	up	2.32	0.44
s__Ruminococcaceae_unclassified	down	-0.78	0.44
s__Anaerofustis_unclassified	up	2.81	0.50
s__Bacillus_cereus	down	-1.48	0.50
s__Family_XIII_AD3011_group_unclassified	up	1.32	0.50
s__Flavobacteriaceae_unclassified	up	2.12	0.50

MP, basal diet + microplastics challenge; MAM, basal diet + 300 mg/kg artemisia deserti polysaccharide + microplastics challenge.

Table S4. Significantly altered bacteria between the MP and HAM groups.

Bacteria	up/down	Log2FC	*P*-value
f__Christensenellaceae	up	1.54	0.46
f__Clostridiaceae	up	2.00	0.46
f__Butyricicoccaceae	down	-1.27	0.46
f__Anaerovoracaceae	up	0.83	0.46
g__Christensenellaceae_R-7_group	up	1.58	0.36
g__Anaerovoracaceae_unclassified	up	1.57	0.36
g__Paraprevotella	up	3.53	0.36
g__GCA-900066575	down	-3.37	0.36
g__Ruminococcaceae_unclassified	down	-1.14	0.46
g__Candidatus_Arthromitus	up	2.16	0.46
g__Prevotellaceae_UCG-001	up	0.88	0.46
s__Christensenellaceae_R-7_group_unclassified	up	1.58	0.38
s__Anaerovoracaceae_unclassified	up	1.57	0.38
s__Paraprevotella_unclassified	up	3.53	0.38
s__GCA-900066575_unclassified	down	-3.37	0.38
s__Ruminococcaceae_unclassified	down	-1.14	0.45
s__Candidatus_Arthromitus_unclassified	up	2.16	0.45
s__Prevotellaceae_UCG-001_unclassified	up	0.88	0.45

MP, basal diet + microplastics challenge; HAM, basal diet + 450 mg/kg artemisia deserti polysaccharide + microplastics challenge.
